# Sustainability through Biomimicry: A Comprehensive Review of Bionic Design Applications

**DOI:** 10.3390/biomimetics9090507

**Published:** 2024-08-23

**Authors:** Mu Jiang, Wenxin Deng, Hong Lin

**Affiliations:** School of Art, Soochow University, Suzhou 215123, China; jiangmu@suda.edu.cn

**Keywords:** bionic design, sustainable development, industrial design, ecological integration, technological innovation

## Abstract

The research objective of this paper is to examine the role of bionic design in advancing sustainable development within industrial design by outlining its theoretical framework; analyzing its applications in morphological, functional, and material aspects; identifying current challenges; and projecting future trends toward eco-integration, resource efficiency, and technological innovation. First, the definition, development history, and theoretical basis of the sustainable development of bionic design are outlined. Secondly, the application of bionic design in sustainable industrial design is analyzed in depth, including the application of morphological bionic design in exploring the combination of nature and innovation, the role of functional bionic design in integrating biological function and product innovation, and the harmonious unification of material bionic and environmental friendliness. Finally, it points out the current challenges faced by bionic design, such as barriers in design practice and market acceptance issues, and looks forward to the sustainable development trend of bionic design, including eco-integration, resource efficiency enhancement, technological innovation, integrated application, etc., to provide new ideas and impetus for the sustainable development of the industrial design field in the future.

## 1. Introduction

In today’s world, environmental problems and resource constraints have become global challenges as industrialization continues [1]. Against this background, the concept of sustainable development has emerged, which emphasizes meeting the needs of the present without compromising the ability of future generations to meet their needs [2]. Industrial design, as an important force to promote social progress and improve human life, carries the important mission of realizing sustainable development. In this process, bionic design, as an innovative design method, has received widespread attention due to its unique value and potential. Bionic design, also known as biomimetic design, provides inspiration and solutions for industrial product design by imitating the form, structure, function, and other characteristics of creatures in nature [3]. Nature’s creatures have evolved over hundreds of millions of years to develop characteristics such as high efficiency, energy saving, and adaptability, which are highly compatible with the goal of sustainable development. Therefore, applying bionic design to industrial design can not only enhance the innovation and practicality of products but also promote the greening and decolonization of design, providing new ideas and methods to realize the sustainable development of industrial design.

However, although the application of bionic design in industrial design is becoming more and more widespread, and it can be seen in various fields, from electronic products and furniture design to architecture and transportation, there are still many challenges and problems in how to more effectively combine the principles of bionic design with the goal of sustainable development, and how to systematically apply bionic design in design practice [4]. For example, how to ensure that bionic design does not only stay at the level of morphological imitation but goes deeper, into functional and structural innovation, how to assess the environmental and social benefits of bionic design products, and how to establish a set of perfect bionic design methodology and evaluation system.

The analysis of bionic design principles involves a comprehensive study that encompasses the examination of nature’s models, the abstraction of their functional principles, and the translation of these principles into innovative design solutions that address current technical and ecological challenges. This approach not only seeks to understand the structural and functional intricacies of biological systems for technological advancement but also aims to align human-made designs with the sustainability and resilience found in nature. In essence, the analysis of bionic design principles is an interpretive process that draws from the natural world to inspire solutions that are not only innovative but also inherently compatible with environmental sustainability.

Based on this, by systematically analyzing the principles, methods, and practice cases of bionic design, this study proposes a set of practical application strategies to provide theoretical and practical guidance for the sustainable development of the industrial design field. The study will not only enrich the theoretical system of bionic design and provide new theoretical perspectives and methodologies for industrial design, but it will also provide designers with specific application strategies and tools to help them better integrate bionic design concepts into product design, promote design innovation, and improve the ecology and sustainability of products. At the same time, it will also provide references and lessons for the application of bionic design in other fields, promote interdisciplinary exchanges and cooperation, and jointly promote the sustainable development of the community of human destiny [5].

## 2. Bionic Design and Sustainable Development

### 2.1. Definition and Development of Bionic Design

The concept of bionics, an interdisciplinary field, was first proposed by the Austrian scholar Erasmus Stradivarius in the late 19th century [6]. Stradivarius defined bionics as “the science of solving human technical problems by imitating the form and function of natural organisms”. This definition not only reveals the core idea of bionics, which is to draw inspiration from nature, but also points to its wide range of applications: the forms and functions of organisms in nature can provide an endless source of inspiration for human technological innovation. The core of bionics lies in the observation and study of organisms in nature, including their structures, functions, behaviors, and survival strategies, and the extraction of useful principles and mechanisms that can be applied to a variety of technological problems in human society [7]. This innovative approach to mimic the natural world not only helps us design more efficient, economical, and sustainable technological solutions but also promotes our understanding of and respect for the natural world, which in turn promotes the harmonious coexistence of human society and the natural environment [8].

The history of bionic design, as the application of bionics in the field of industrial design, can be traced back to the early stages of human civilization [9]. As early as in ancient times, people had begun to imitate creatures in nature to design and manufacture tools [10]. For example, ancient boat designs mimicked the shape of a fish to improve their navigational efficiency in the water, while ancient buildings mimicked the structure of a bird’s nest to enhance its stability and durability. However, the development of bionic design as a systematic discipline occurred mainly after the mid-20th century, when engineers in the U.S. Air Force first coined the term “bionics” in 1958 and began to systematically investigate how biological principles from nature could be applied to the design of aviation technology [11]. Subsequently, this concept was rapidly expanded to other areas of industrial design, and in the 1960s, with the rapid development of technology and growing concern for the ecological environment, bionic design began to be favored by more designers and engineers. Designers began to study more deeply the form and function of living creatures in nature and tried to apply these principles to the design of various industrial products [12]. For example, lightweight materials that mimic the structure of a honeycomb and high-performance fibers that mimic the strength of a spider’s silk are important achievements of bionic design in this period. Entering the 21st century, with the development of computer-aided design (CAD) and computer-aided manufacturing (CAM) technology, bionic design has ushered in new development opportunities [12]. Designers can more accurately simulate biological forms and structures in nature and apply these complex forms to product design. In addition, with the emphasis on sustainable development, bionic design is increasingly being applied to green design and eco-design to achieve environmentally friendly and sustainable products [13]. In the past decades, the application of bionic design in industrial design has made remarkable achievements. From airplane designs that mimic the flight principles of birds to self-cleaning surface technologies that mimic the lotus effect, as well as lightweight and high-strength materials that mimic the structure of the human skeleton, bionic design has brought us many innovative and efficient design solutions. These achievements not only improve the functionality and aesthetics of products but also help to reduce resource consumption and environmental pollution and promote the sustainable development of industrial design.

However, despite the progress made in the application of bionic design in industrial design, it still faces many challenges and problems [8], for example, how to understand and mimic complex biological systems in nature more deeply, how to apply bionic design principles more widely in the design of various industrial products, and how to establish and improve the evaluation system of bionic design [14]. The solution to these problems requires the joint efforts and cooperation of multidisciplinary experts such as designers, engineers, biologists, and environmental scientists.

### 2.2. Theory and Development of Sustainable Development

The theory of sustainable development is a comprehensive concept that has its origins in the issue of harmonizing environmental protection with economic development [15]. The core of this theory lies in realizing economic growth while also protecting environmental resources and ensuring social equity in order to meet the needs of the present generation without compromising the ability of future generations to meet their needs [16]. The concept can be traced back to the 1970s, when, as global environmental problems became more and more pronounced, mankind began to reflect on the negative impacts of the traditional development model. The year 1987 saw the release of a landmark report by the UN’s World Commission on Environment and Development (WCED)—a report on the impacts of economic growth on the environment. In 1987, the UN World Commission on Environment and Development (WCED) released a landmark report, *Our Common Future*, which was prepared under the leadership of former Norwegian Prime Minister Gro Harlem Brundtland and, for the first time, explicitly proposed the definition of “sustainable development”. The report, prepared under the leadership of former Norwegian Prime Minister Gro Harlem Brundtland, was the first to clearly define “sustainable development” as “meeting the needs of the present without compromising the ability of future generations to meet their own needs”. This definition laid the foundation for subsequent environmental policies and international development strategies and had a profound impact on the global sustainable development agenda [17]. Entering the 1990s, the theory of sustainable development was further developed and refined as global climate change, loss of biodiversity, resource depletion, and other problems intensified. In 1992, the United Nations Conference on Environment and Development (UNCED) was held in Rio de Janeiro, Brazil, and the conference adopted the Rio Declaration and Agenda 21. The two documents emphasized the shared responsibility of governments in promoting sustainable development and proposed a series of concrete action plans and targets [18].

**Figure 1:**
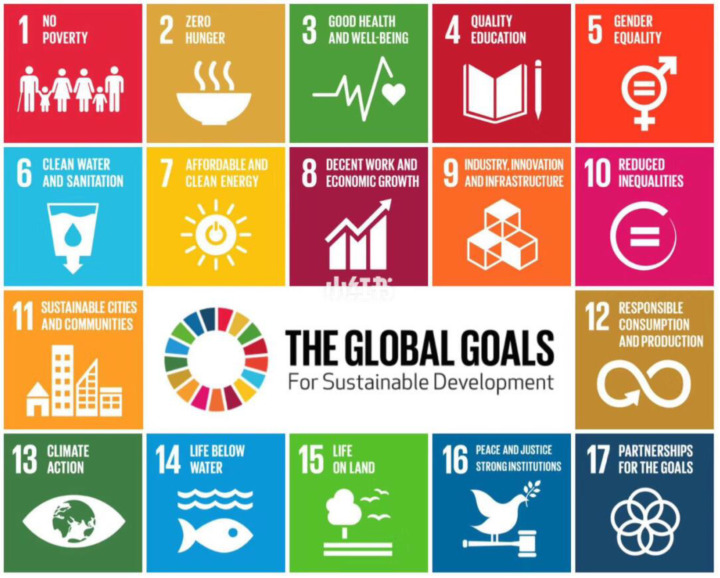
The 17 United Nations’ sustainable development goals (http://www.citmt.cn/news/201910/60476.html (accessed on 27 May 2024)).

At the beginning of the 21st century, the theory of sustainable development was further expanded to include social, economic, and environmental dimensions, resulting in the so-called “Triple Bottom Line” (Triple Bottom Line), i.e., economic prosperity (profit), social inclusion (people), and environmental responsibility (planet). This concept emphasizes that any development activity must balance these three dimensions to ensure long-term sustainability [19]. In 2015, the United Nations adopted the 2030 Agenda for Sustainable Development (“Transforming Our World: The 2030 Agenda for Sustainable Development”), which contains 17 Sustainable Development Goals (SDGs) that aim to address poverty, inequality, climate change, and other issues on a global scale, and to promote global sustainable development (Figure 1). These goals cover a wide range of areas, from poverty eradication and zero hunger to clean energy, inequality reduction, and climate action, and provide a comprehensive and coordinated framework for global sustainable development [20].

The development of the theory of sustainable development has not only promoted the international community’s attention to environmental issues but also facilitated changes in policy formulation and implementation in various countries. Under the guidance of this theory, many countries have begun to incorporate the principles of sustainable development into their national development strategies and to promote the development of green, circular, and low-carbon economies [21]. At the same time, enterprises and social organizations have begun to actively participate in the practice of sustainable development, contributing to the realization of sustainable development goals through technological innovation, management optimization, and social responsibility practices.

However, the practice of sustainable development also faces many challenges [22]. Firstly, there are differences in the development stages, resource endowments, and cultural backgrounds of different countries and regions, thus making it necessary to tailor the paths and strategies of sustainable development to local conditions. Secondly, sustainable development requires long-term and systematic planning and investment, while short-term economic interests often conflict with long-term environmental goals, thus requiring policymakers and all sectors of society to make wise choices on the balance of interests. In addition, global sustainable development issues, such as climate change and biodiversity conservation, require the collaboration and joint efforts of the international community, and there are still many uncertainties and instabilities in the current international cooperation mechanism. Looking ahead, the theory of sustainable development will continue to develop and deepen. With the progress of science and technology and the improvement of social awareness, people will pay more attention to the realization path of sustainable development and the evaluation of its effects. Emerging technologies, such as big data, artificial intelligence, and renewable energy, will provide new ideas and tools for sustainable development. At the same time, interdisciplinary and cross-field cooperation will become an important way to promote sustainable development, creating synergies through the integration of knowledge and resources from different fields to jointly address global challenges.

## 3. Analysis of the Application of Bionic Design in Sustainable Industrial Design

### 3.1. Morphological Bionic Design: Exploring the Intersection of Nature and Innovation

The theoretical foundation of biomimetic design is deeply rooted in the profound discipline of bionics. Bionics is a discipline that specializes in the study of the structure and function of biological systems and tries to draw inspiration from them to solve the technological challenges faced by human beings [23]. After billions of years of evolution, natural organisms have demonstrated a high degree of adaptability and superiority in terms of form, structure, and function. These characteristics undoubtedly provide a rich source of inspiration for designers. The innovative value of biomimetic design lies in its ability to bring unprecedented innovation to product design. Through the imitation of natural biological forms, designers can break the traditional design limitations and create products that meet people’s aesthetic needs, as well as practical-use needs. At the same time, biomimetic design can also improve the functionality and practicality of the products, so that the products can be more humanized and intelligent, based on meeting the basic needs of use. In addition, morphological bionic design also has a high aesthetic value. Biomorphic forms in nature, whether complicated insect wings or simple bird feathers, all contain high aesthetic elements. By imitating these biological forms, designers integrate these aesthetic elements into product design, making the product not only of practical value but also a work of art to meet people’s pursuit of beauty. More importantly, biomimetic design also has important environmental and energy-saving values. Through the study and imitation of natural biological forms, designers can better understand the relationship between organisms and the environment and, thus, pay more attention to environmental protection and energy saving when designing products. For example, by mimicking the photosynthesis of plants, they can design products that can supply their power; or by mimicking the morphology and structure of animals, they can design more energy-saving transportation.

The process of biomimetic design is not a quick fix, and it requires designers to have a deep interdisciplinary knowledge background and keen insights. First of all, designers need to observe and study the living creatures in the natural world to understand their morphology, structure, functional characteristics, and living environment. Then, they need to abstract and refine the key design elements from this information and combine them with the product’s functional requirements and usage scenarios to create innovative designs. During the design process, designers need to constantly test and improve to ensure that the functionality and aesthetics of the product are perfectly combined.

**Figure 2:**
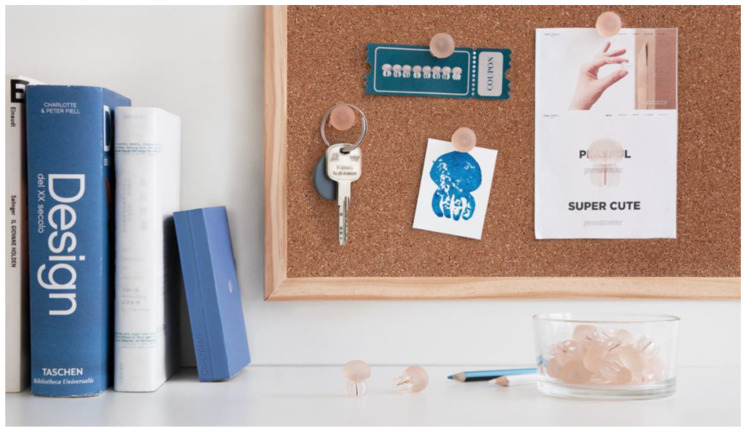
This scenario of the “Jellyfish” pin (http://k.sina.com.cn/article_2480472101_93d90025019018npp.html (accessed on 27 May 2024)).

The practical application of morpho-bionic design has been involved in many fields [24]. In the field of transportation design, designers have designed lighter and more efficient flying machines by imitating the wing structure of birds; in the field of architectural design, designers have designed more ventilated and light-permeable buildings by imitating the arrangement of leaves; in the field of apparel design, designers have designed more fashionable and personalized clothing styles by imitating the fur texture and color combination of animals; and in the field of daily necessities, designers need to constantly experiment and improve to ensure the perfect combination of functionality and aesthetics of products. In the field of clothing design, designers imitate animal fur textures and color combinations to create more fashionable and personalized clothing styles; in the field of daily necessities, designer Minjuan Yao designed a “bionic jellyfish nail” based on the jellyfish form (Figure 2). Taking the jellyfish as the prototype, the overall shape of the nail is outlined by simple and smooth lines, making it as light and elegant as a jellyfish; the environmentally friendly and recyclable plastic material is chosen, and through a special process, the surface of the nail shows a transparent or translucent texture, which is as if it looks like a jellyfish cruising in the water; it not only embodies the unique charm of the morphology of bionic design but also provides useful insights into the environmental protection and sustainable development of the world. This not only reflects the unique charm of morpho-bionic design but also provides useful inspiration for environmental protection and sustainable development.

Despite the many advantages and potentials of biomimetic design, it also faces some challenges in practical application. Firstly, there are certain differences and limitations between biomorphic and man-made products, which require designers to make appropriate adjustments and improvements when designing. Secondly, there are certain differences in performance between biomaterials and man-made materials, which will also bring certain difficulties to the realization of design solutions. In addition, morpho-bionic design requires designers to have an interdisciplinary knowledge background and innovation ability, creating a considerable challenge.

### 3.2. Functional Biomimetic Design: Uncovering the Integration of Biological Functions and Product Innovation

Functional biomimetic design, as the name suggests, is a method of product design that imitates the functional mechanism of living organisms. It extracts the principles and laws from the in-depth study of the functions of organisms, such as movement, perception, and energy conversion, and applies them to product design to realize the improvement and innovation of product performance [25]. This design method emphasizes the in-depth understanding and imitation of biological functions, aiming to transform the natural advantages of living organisms into the technical advantages of products to realize product innovation and development. In functional bionic design, the functional mechanisms of living organisms are the source of design inspiration. These mechanisms have evolved over billions of years to form efficient, stable, and adaptable features, which provide rich references for product design. For example, the swimming style of fish, the flying skills of birds, and the jumping ability of insects provide optimization ideas for the motion performance of products; the path-planning ability of ants and the echolocation ability of bats inspire the intelligent control of products; and the water storage ability of cacti and the water-saving mechanism of desert plants provide references for energy-saving design of products.

**Figure 3:**
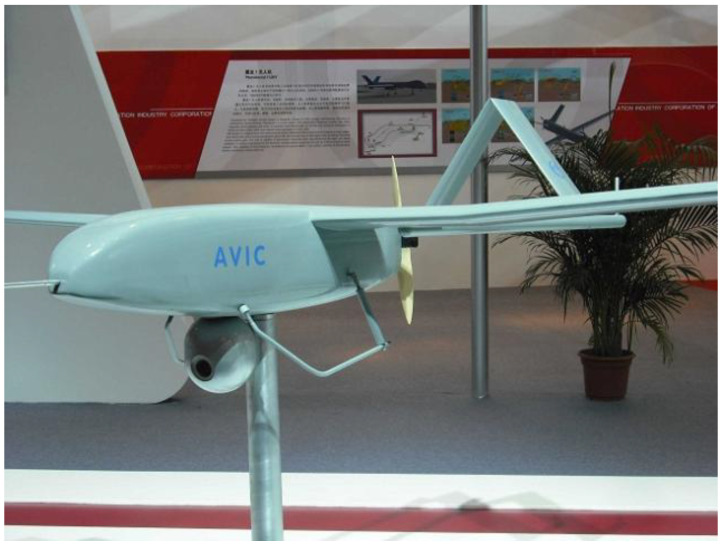
Nighthawk UAV navigation system (https://www.newton.com.tw/wiki/%E5%A4%9C%E9%B7%B9%E7%84%A1%E4%BA%BA%E6%A9%9F (accessed on 27 May 2024)).

**Figure 4:**
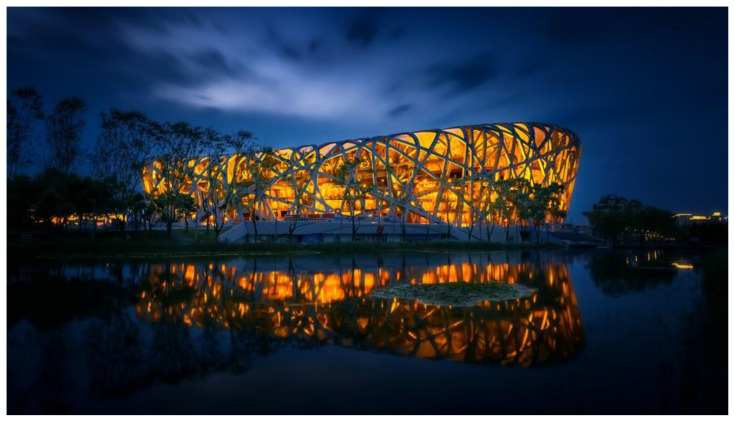
Design of the Bird’s Nest, the main stadium of the Beijing Olympics (http://www.hbgmjs.com/front/page/138648/article_id/38786.html (accessed on 27 May 2024)).

The application of functional bionic design in product innovation is mainly in the following ways. First, motion bionic design: The movement mechanism of living organisms is an important research object of functional bionic design. By imitating the motion of living organisms, designers can optimize the motion performance of products and improve their working efficiency in specific environments. For example, an underwater vehicle designed to mimic the swinging of a fish’s fin can achieve a more efficient and flexible propulsion method, while a vehicle designed to mimic the flapping of a bird’s wing can be optimized in terms of aerodynamic performance. In addition, the jumping ability of insects also provides new ideas for product design, such as jumping robots designed to mimic the jumping mechanism of locusts, which can move freely in complex terrains, providing convenience in areas such as exploration and rescue. Second, perception bionic design: The perception ability of organisms is also an important research object of functional bionic design. By imitating the perception mechanism of living organisms, designers can design more intelligent and sensitive products. For example, the path-planning ability shown by ants when searching for food inspires the development of logistics optimization algorithms, and the echolocation ability of bats inspires the design of radar systems. The mimicry of these intelligent behaviors makes products more efficient and flexible in dealing with complex problems. In addition, the visual, auditory, olfactory, and other perceptual abilities of living organisms also provide new ideas for product design. For example, the design of China’s self-developed Nighthawk UAV navigation system was inspired by the excellent night-vision capabilities of the Nighthawk (owl). These perceptual abilities of owls are applied to modern navigation technology, especially to provide assistance for driving at night or under low visibility conditions (Figure 3). Not only does it embody the concept of perceptual bionic design, but it also demonstrates how the biological properties of nature can be transformed into modern technologies to solve real-world problems. Third, energy conversion bionic design: Living organisms also have high efficiency in energy conversion. By mimicking the energy conversion mechanism of living organisms, designers can design more efficient and environmentally friendly energy utilization systems. For example, the water storage capacity of cacti and the water-saving mechanism of desert plants provide references for water resource utilization; solar panels imitating plant photosynthesis can convert solar energy into electricity more efficiently. These designs not only improve the efficiency of energy utilization but also reduce the negative impact on the environment. For example, the design of the Bird’s Nest, the main stadium for the 2008 Beijing Olympics, has a roof design that makes full use of natural light and reduces reliance on artificial lighting. In addition, the building utilizes energy-efficient materials and equipment to reduce energy consumption. Its unique design and environmental protection concepts have won wide acclaim and become one of the models of modern architectural design (Figure 4).

Although functional bionic design has shown great potential in product innovation, it still faces several challenges in its practical application. First, the functional mechanisms of living organisms are often very complex and diverse, requiring in-depth interdisciplinary research to fully understand and mimic. This requires designers to have a broad knowledge base and the ability to innovate. Secondly, translating the principles of biological function into practical engineering applications requires a high degree of innovation and engineering support. Designers need to acquire knowledge and skills in the fields of advanced materials science, mechanical engineering, and information technology to achieve this goal.

### 3.3. Material Bionics and Environmental Friendliness: Exploring the Harmonious Integration of Nature Inspiration and Sustainable Innovation

Material bionic design is a method of designing and manufacturing new materials by simulating the structure and function of biological materials, drawing inspiration from nature. Biomaterials in nature have evolved over billions of years to develop unique structures and functions that not only provide excellent performance but also maintain stability and reliability in complex and changing environments [26]. Therefore, material bionic design emphasizes in-depth understanding and research of biological materials in nature, as well as the application of these research results in material design and manufacturing. In the process of material biomimetic design, typical and representative biomaterials need to be selected as research objects first. These biomaterials usually have unique structures and functions, such as the strength and toughness of spider silk, the self-cleaning function of lotus leaves, and the strength and toughness of shells. Through the in-depth research and analysis of the structure and function of these biomaterials, the laws and principles embedded in them can be discovered, providing inspiration and a basis for the design of new materials. In the process of material biomimetic design, it is also necessary to use modern scientific and technological means, such as nanotechnology, biotechnology, etc., to simulate and replicate the structure and function of biomaterials. By simulating the microstructure and molecular composition of biomaterials, new materials with similar properties can be developed. At the same time, by introducing new elements and compounds, the properties of the materials can be further improved and optimized to make them more in line with the needs of practical applications. The advantage of material bionic design is that new materials with excellent properties can be developed. These materials not only have excellent properties, such as high strength, high toughness, and wear resistance, but they also have unique functions and properties, such as self-healing ability and environmental responsiveness. These excellent properties give the material bionic design a wide range of application prospects in many fields, such as aerospace, automobile manufacturing, biomedical, and so on.

**Figure 5:**
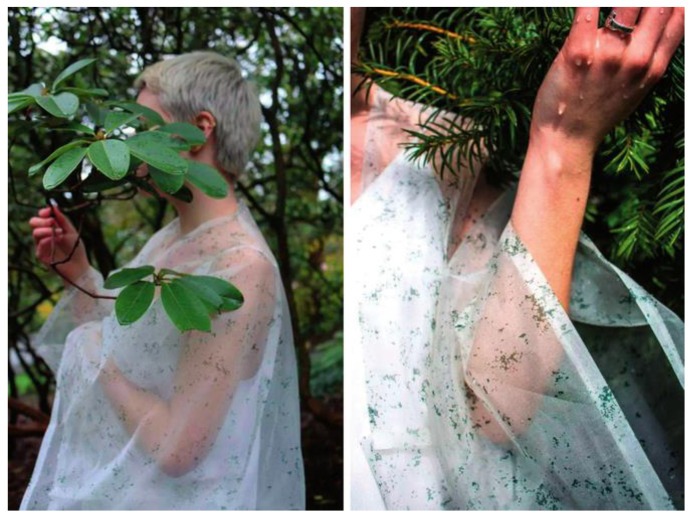
Algae-based jacket “Biogarmentry” design (https://baijiahao.baidu.com/s?id=1649251538779148184&wfr=spider&for=pc (accessed on 27 May 2024)).

The environmentally friendly design aims to achieve a minimal negative impact on the environment throughout the life cycle of materials, products, and systems [27]. This design philosophy emphasizes the need to fully consider the environmental impact of materials in all aspects of their design, manufacture, use, and recycling, and to take measures to reduce the negative impact. Environmentally friendly design can not only reduce resource and energy consumption and waste generation but also improve the recyclability and reuse of materials, thus realizing the recycling of resources and sustainable development. The importance of environmentally friendly design is self-evident. As global environmental problems become more and more serious, mankind must take action to reduce its impact on the environment. In the field of materials science, environmentally friendly design can promote the green manufacturing and recycling of materials and reduce the damage and pollution to the environment. At the same time, environmentally friendly design can also promote the research and development of new materials and technologies and promote the transformation and upgrading of related industries and sustainable development. To realize environmentally friendly design, a series of measures need to be taken. Firstly, in the process of material design, it is necessary to give full consideration to the impact on the environment during the source, manufacture, and use of materials and to select materials with low impact on the environment. Secondly, in the process of material manufacturing, green manufacturing techniques and technologies should be adopted to reduce energy consumption and waste emissions. In addition, in the process of material use, it is necessary to adopt reasonable use and maintenance measures to extend the service life of materials. Finally, in the process of material recycling and reuse, it is necessary to establish a perfect recycling system and reuse mechanism to realize the recycling of resources. For example, Canadian designer Roya Aghaii has designed a garment that uses algae to photosynthesize to convert carbon dioxide into oxygen, called Biogarmentry, which is made by spinning BioRhine Chlamydomonas (a unicellular green alga), together with nano-polymers, to create a lightweight woven eco-textile similar to linen, which can photosynthesize like a plant (Figure 5).

The fusion of material biomimicry and environmentally friendly design is a design concept that combines nature-inspired design with sustainable innovation. This fusion of design concepts emphasizes the importance of considering both the performance and functional needs of materials and their environmental friendliness in the material design process. By mimicking the structure and function of biomaterials, new materials with excellent properties can be developed, and environmentally friendly design ensures that the environmental impact of these materials is minimized during production and use. In the process of integrating materials biomimicry and environmentally friendly design, several factors need to be considered comprehensively. Firstly, typical and representative biomaterials should be selected as research objects, and their structures and functions should be studied in depth. Secondly, the structure and function of biomaterials should be simulated and reproduced by modern technology, and new materials with similar properties should be developed. At the same time, in the process of material design, the environmental friendliness of the material should be fully considered, and materials and manufacturing processes with low impact on the environment should be selected. Finally, in the process of material use, reasonable use and maintenance measures should be taken to ensure that the environmental friendliness of the material is fully realized. This integrated design concept has a wide range of application prospects. In the construction industry, new building materials with high strength, high toughness, and good thermal insulation performance can be developed through material bionic design; at the same time, the recyclability and reuse of building materials can be realized through environmentally friendly design to reduce the generation of construction waste. In the field of energy, material bionic design can develop new energy materials with efficient energy conversion and storage performance, while environmentally friendly design can ensure that these materials are in the production and use of the process of minimizing the impact on the environment. In addition, in the fields of healthcare, transportation, and electronics, the integration of material bionics and environmentally friendly design will also play an important role in promoting the sustainable development of related industries.

## 4. Challenges Facing Bionic Design and Sustainable Development Trends

### 4.1. Challenges Faced by Bionic Design at Present

With the continuous progress of science and technology and mankind’s desire for a deeper understanding of the natural world, bionic design has gradually become an important branch of the design field. Bionic design, as the name suggests, is a method of design that imitates the structure, function, form, and other characteristics of living organisms, aiming to create products that meet human needs and have a sense of natural beauty at the same time. However, despite the many advantages of bionic design, it still faces many challenges in the process of practical application and marketing.

#### 4.1.1. Subsubsection

In design practice, the obstacles faced by bionic design are multifaceted. First, the complexity of living organisms brings great challenges to design. Through billions of years of evolution, organisms have formed highly complex, fine, and coordinated structures and functions. When these complex biological features are transformed into design elements, designers are required to have profound biological knowledge, design skills, and innovation ability. However, at present, many designers do not have enough knowledge of biology, and it is difficult for them to deeply understand and imitate the characteristics of living organisms, thus leading to the lack of realism and biological sense in the designed products. Secondly, bionic design requires numerous factors to be considered in the design process. In addition to the structure, function, and form of living organisms, it is also necessary to consider the use of the product environment and the use of the population, production costs, and other factors. The comprehensive consideration of these factors requires designers to have interdisciplinary knowledge reserves and comprehensive capabilities. However, at present, many designers have limited ability in terms of interdisciplinary aspects, and it is difficult to consider various factors comprehensively, thus leading to many problems in the practical application of the designed products. In addition, bionic design also needs to overcome technical difficulties in the design process. Many features of living organisms are technically difficult to realize, such as the self-adaptation and self-repair capabilities of living organisms. These technical difficulties require designers to have profound knowledge of engineering technology and material science, as well as the spirit of continuous innovation. However, at present, many designers have limited ability in regard to these aspects, resulting in bionic design being technically difficult or costly to realize [28].

#### 4.1.2. Market Acceptance and Consumer Perception

In addition to the barriers in design practice, bionic design also faces the challenges of market acceptance and consumer perception [29]. First of all, since bionic design is an emerging design method, its design concept and design results have relatively low recognition in the market. Many consumers do not understand the concept, characteristics, and advantages of bionic design, thus leading them to prefer traditional design products when purchasing products. This lack of market awareness limits the marketing and sales of bionic design products.

Secondly, the market acceptance of bionic design products is also affected by various factors. On the one hand, because the design concepts and design results of bionic design products are quite different from those of traditional design products, it leads to doubts and concerns of some consumers when they buy them. They worry that there are problems with the performance, quality, and reliability of bionic design products and thus choose traditional design products. On the other hand, the higher production cost of bionic design products leads to their relatively higher selling price. This price disadvantage discourages some consumers from making purchases, thus limiting the market acceptance of bionic design products.

### 4.2. Sustainable Strategies for Bionic Design

#### 4.2.1. Ecological Integration and Environmental Symbiosis

**Figure 6:**
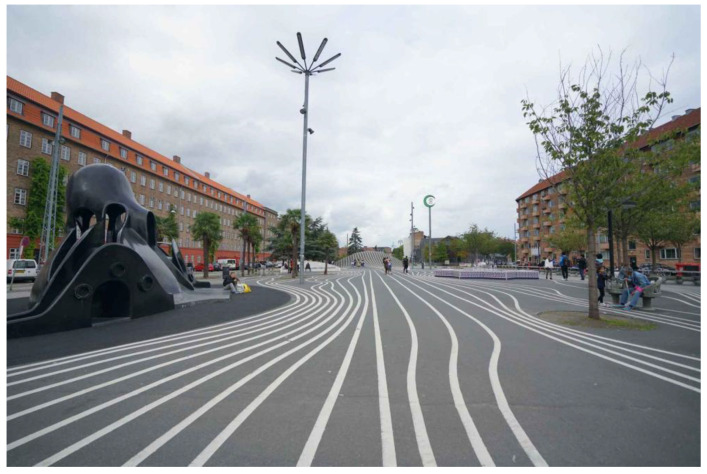
Super Linear Park, Copenhagen (https://www.sohu.com/a/364839160_111938 (accessed on 27 May 2024)).

In the face of today’s increasingly serious environmental problems, the design field is actively exploring design concepts and methods for harmonious coexistence with nature. Bionic design, as one of the potential design concepts, is centered on the design and innovation of artificial systems based on the form, structure, and functional characteristics of living organisms. This design concept not only reflects the respect and reverence for the natural ecology but also realizes the deep integration and symbiosis between the artificial system and the natural environment by simulating the survival strategy of living organisms. The ecological integration of bionic design is reflected in several aspects. First of all, in morphological design, bionic design draws on the unique forms of living organisms, such as the feathers of birds, the wings of insects, etc. By mimicking these natural forms, it creates design works that are both aesthetically pleasing and in line with ecological principles. For example, in architectural design, the Bird’s Nest Gymnasium, which imitates the structure of a bird’s nest, not only has a unique appearance but also has a light structure and reasonable force, effectively reducing the burden of the building on the environment. Secondly, in structural design, the bionic design utilizes the structural principles of living organisms, such as the hexagonal structure of the beehive, the elastic structure of the spider’s web, etc., to design structural systems with high strength, high stability, and high adaptability. These structural systems excel in resisting natural disasters, bearing heavy loads, etc., enabling artificial systems to remain stable and safe under various environmental conditions. Taking bridge design as an example, bridges inspired by bionic design, by stimulating the growth of plant roots and the mechanical properties of spider webs, design bridge structures with a strong load-bearing capacity and good seismic performance, realizing the harmonious coexistence of the artificial system and the natural environment. Furthermore, in terms of functional design, bionic design gives stronger self-adaptation and ecological restoration ability to the artificial system by simulating the physiological functions of living organisms, such as the sensing system of animals and photosynthesis of plants. In urban planning, the use of bionic design concepts makes urban space more in line with natural ecological laws, such as through the design of rain gardens that simulate the natural water circulation system, effectively improving the water environment and microclimate of the city. In addition, biomimetic design also emphasizes the symbiotic relationship with the environment. In material selection, bionic design tends to use renewable and biodegradable environmental materials to reduce the consumption of natural resources and pollution of the environment. In energy utilization, bionic design focuses on the use of solar energy, wind energy, and other clean energy types to improve energy efficiency and reduce energy consumption. In waste treatment, bionic design advocates for the recycling of resources and harmless treatment of waste to reduce the negative impact on the environment [30]. Taking the super-linear park in Copenhagen, Denmark, as an example (Figure 6), we can see that the project fully embodies the ecological integration and environmental symbiosis concept of bionic design. The project integrates urban space with the natural environment by simulating the structure and function of the natural ecosystem. There are several ecological restoration zones in the park that utilize natural processes such as the photosynthesis of plants and decomposition of microorganisms to restore and improve environmental elements, such as soil and water. At the same time, the park also adopts a variety of renewable energy technologies, such as solar photovoltaic power generation and wind power generation, to provide a green and low-carbon energy supply for the park. In addition, the park has set up waste-recycling and -treatment facilities to realize the recycling of resources and the harmless treatment of waste.

The concept of ecological integration and environmental symbiosis of bionic design has brought new ideas and methods to the design field. By drawing on the morphological, structural, and functional characteristics of living organisms, bionic design creates design works that are both aesthetically pleasing and in line with ecological principles, realizing the deep integration and symbiosis between artificial systems and the natural environment. In future development, biomimetic design will continue to play an important role in promoting the harmonious coexistence of man and nature, contributing wisdom and strength to the sustainable development of human society.

#### 4.2.2. Resource Efficiency and Circular Economy

At a time when resources are becoming increasingly scarce and environmental pressure is intensifying, biomimetic design, with its unique perspective and strategy, opens up a new way to enhance resource efficiency and promote the development of a circular economy [31]. Bionic design simulates the operation of natural ecosystems through an in-depth study of efficient resource utilization strategies and recycling mechanisms in the biological world and integrates them into all aspects of product design, manufacturing, and management to maximize the use of resources and minimize the emission of wastes, thus promoting the sustainable development of the economy and society. In terms of resource efficiency, bionic design digs deep into the structural and functional characteristics of living organisms, providing valuable inspiration for product design. In architectural design, bionic design can draw on the photosynthesis principle of plants, and by optimizing the building layout and using highly efficient photovoltaic materials and green building materials, it can significantly improve the efficiency of the building’s use of solar energy and reduce its dependence on traditional energy sources. At the same time, bionic design also focuses on the lightweight design of products, through precise calculation and optimization of the material structure, reducing unnecessary material consumption and lowering the energy consumption and carbon emissions of products. In transportation design, bionic design imitates the flight principle of birds and the crawling mechanism of insects to design more energy-saving and efficient transportation, such as electric cars and hybrid cars, to reduce energy consumption and environmental pollution.

**Figure 7:**
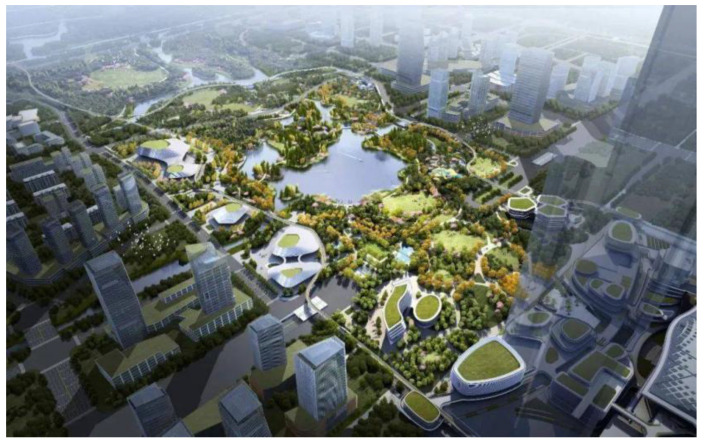
Aerial view design of Yunmen Park, a world-class city park in Hangzhou (http://zzhz.zjol.com.cn/hz/csxw/202303/t20230309_25507013.shtml (accessed on 27 May 2024)).

In terms of circular economy, bionic design emphasizes the recycling of resources and the harmless treatment of waste. By simulating the cyclic regeneration mechanism of the natural ecosystem, bionic design treats waste as a valuable resource and transform it into new products or energy using classification, recycling, and reuse. In the product-design stage, bionic design actively adopts biodegradable and renewable environmentally friendly materials to reduce the impact of products on the environment. Meanwhile, through modularized design, it is easy to maintain and upgrade the products, extend the service life of the products, and reduce the waste of resources. In the manufacturing process, bionic design advocates for the use of clean production technology to reduce the generation and emissions of waste. In terms of waste treatment, bionic design introduces advanced technologies such as biodegradation and heat recovery to convert waste into new resources or energy and realize the recycling of resources. Take the example of Hangzhou Yunmen Park (Figure 7). First, the park draws on the cyclic regeneration mechanism of natural ecosystems in its planning and realizes the green ecological cycle through the rational arrangement of vegetation and the construction of ecological trails. Second, the facilities in the park adopt energy-efficient materials and technologies, such as solar photovoltaic power generation and rainwater collection systems, which significantly increase the efficiency of resource utilization. Finally, the design of Yunmen Park not only considers environmental sustainability but also emphasizes the recycling of resources, such as waste separation and recycling, ecological restoration, and other measures that effectively reduce environmental pressure.

#### 4.2.3. Technology Innovation and Integrated Application

At a time of global resource constraints and increasing environmental challenges, bionic design, with its unique innovation strategy, has become an important force in promoting resource efficiency and the circular economy [32]. Bionic design not only imitates the form, structure, and function of natural organisms but also draws on the survival wisdom of organisms, aiming to provide effective solutions to the environmental problems faced by human beings through technological innovation and integrated application. This paper discusses the strategic significance of bionic design and its application in practice from the aspects of technological innovation and integrated application.

Technological innovation is the core driving force for the development of bionic design. Bionic design explores the unique structure and function of living organisms through the in-depth study of their mechanisms and how they adapt to the environment and realize efficient use of resources and recycling. In this process, bionic design draws on the achievements of many disciplines, such as biology, physics, chemistry, and materials science, to develop new technologies and materials by combining the wisdom of nature and the innovative wisdom of human beings. First, bionic design has achieved remarkable results in material innovation. By mimicking the material composition and structure of living organisms, bionic design has developed new materials with excellent performances. For example, by mimicking the strength and toughness of spider silk, researchers have developed high-strength synthetic fibers, and by mimicking the laminated structure of shells, composite materials with excellent impact resistance have been developed. These new materials not only are higher performing but also tend to have lower energy consumption and a smaller environmental impact, providing strong support for the realization of the efficient use of resources and environmental sustainability. Secondly, bionic design has also made important progress in structural innovation. The structures of living organisms are often highly complex and diverse, and these structures are not only capable of realizing the various functions of living organisms but also of adapting to different environments and challenges. By mimicking the structure of living organisms, bionic design has developed novel structures with excellent performances. For example, by mimicking the structure of a honeycomb, a structure with efficient energy absorption and cushioning properties has been designed, and by mimicking the surface structure of a lotus leaf, a new coating with a self-cleaning function has been designed. These new structures are not only able to realize functions that are difficult to achieve with traditional structures but also tend to have higher resource utilization efficiency and a lower environmental impact. In addition, biomimetic design plays an important role in functional innovation. Organisms have a variety of complex and efficient functions that not only enable them to survive and reproduce but also provide important insights for humans. By mimicking the functions of living organisms, bionic design develops products and systems with new functions. For example, by mimicking the flight mechanism of birds, efficient and energy-saving flying machines have been developed, and by mimicking the swimming mechanism of fish, fast and flexible underwater robots have been developed. These new products and systems not only meet human needs but also tend to have lower energy consumption and less of an environmental impact.

**Figure 8:**
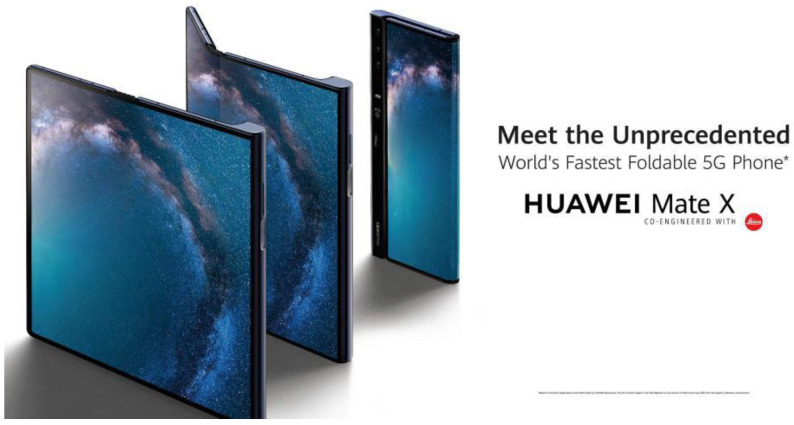
Huawei Mate X series folding-screen phone (https://www.vmall.com/product/10086929639284.html (accessed on 27 May 2024)).

Integrated application is an important way for bionic design to realize its strategic significance. Bionic design not only focuses on the innovation of a single technology but also pays more attention to how to integrate these technologies into actual products and systems to realize their value in practical applications. In terms of integrated applications, bionic design emphasizes interdisciplinary and cross-disciplinary cooperation and integration, creating products and systems with a higher performance and lower environmental impact by integrating knowledge and technologies from different fields. First, in the field of architecture, bionic design realizes the development of green buildings through integrated applications. Green building emphasizes the harmonious symbiosis between buildings and the environment and realizes low energy consumption, low emissions, and high efficiency of buildings by adopting energy-saving and environmentally friendly materials and technologies. Bionic design develops building materials and structures with excellent performance by mimicking the form, structure, and function of living organisms, such as solar photovoltaic panels that mimic photosynthesis in plants and ventilation systems that mimic the structure of insect wings. These new materials and structures not only improve the energy efficiency and comfort of buildings but also reduce the impact of buildings on the environment, providing strong support for the construction of green and sustainable cities. Secondly, in the field of transportation, bionic design promotes the development of environmentally friendly travel through integrated applications. Eco-friendly travel emphasizes the reduction in traffic emissions, the improvement of energy use efficiency, and the improvement of travel experience. Bionic design has developed efficient and energy-saving vehicles and transportation systems by mimicking the movement mechanism and energy-saving principles of living organisms. For example, there are flying machines that mimic the flight mechanism of birds, and there are underwater vehicles that mimic the swimming mechanism of fish. These new vehicles and transportation systems not only improve travel efficiency and comfort but also reduce the impact of transportation on the environment, providing strong support for the realization of green, low-carbon travel. In addition, in other fields, such as energy and manufacturing, bionic design has also realized technological innovation and industrial upgrading through integrated applications. By imitating the energy conversion and storage mechanism of living organisms, bionic design has developed new energy technologies and energy materials, and by imitating the growth and repair mechanism of living organisms, bionic design has realized the intelligence and greening of the manufacturing industry. These technological innovations and industrial upgrades have not only improved the efficiency of resource and energy utilization but also promoted the sustainable development of the industry. Huawei’s Mate X series folding-screen phone, for example, uses a flexible OLED folding screen, which is inspired by bendable and foldable biological structures in nature, such as leaves and wings; the device’s hinge design is similar to the movement mechanism of certain biological joints, making it both lightweight and sturdy. In addition, recyclable materials are used, and a commitment is made to reduce e-waste. This reflects the emphasis on ecosystem preservation in bionic design (Figure 8).

In summary, the above three strategies are interrelated and together form a comprehensive framework aimed at promoting the harmonious coexistence of architecture and the natural environment through bionic design, improving the efficiency of resource utilization, and promoting technological innovation to achieve the goal of sustainable development.

## 5. Conclusions

The world is facing environmental deterioration and resource constraints, and bionic design, as an innovative design concept, can contribute to sustainable development. After an in-depth exploration of bionic design in regard to promoting sustainable development in the field of industrial design, it is not difficult to find that bionic design is not only a bridge that connects nature and innovation but also a key force in promoting industrial design to a greener, more environmentally friendly and sustainable direction. From the natural aesthetics of morphological bionic design to the practical innovation of functional bionic design, and then to the pursuit of environmental friendliness of material bionic design, bionic design has shown great potential and broad application prospects in the field of industrial design. However, bionic design also faces challenges such as design barriers, market acceptance, and consumer perception in the process of practice. These challenges require us to continuously explore and innovate to find more effective solutions. At the same time, we should also see that, with the continuous progress of science and technology and people’s environmental awareness, the sustainable development trend of bionic design is becoming more and more obvious. In response to the pressing environmental and resource challenges of our time, this paper proposes a step-by-step guideline for designers to implement bionic design principles, aiming to navigate the challenges and leverage the potential of bionic design to steer industrial design toward a more sustainable, eco-friendly, and innovative future. Eco-integration, resource efficiency, circular economy, and technological innovation will become important directions for the development of bionic design in the future. Looking ahead, we have reason to believe that bionic design will play a more important role in the field of industrial design, promote the harmonious coexistence of industrial design and the natural environment, and create a better future for mankind.

## Data Availability

The original contributions presented in the study are included in the article, further inquiries can be directed to the corresponding authors.
